# An Atypical Presentation of Extrapulmonary Sarcoidosis

**DOI:** 10.1155/2020/8840245

**Published:** 2020-06-25

**Authors:** Muhammad Shabbir Rawala, Amna Saleem Ahmed, Kristen Helmick

**Affiliations:** ^1^Department of Internal Medicine, WVU-Charleston Division, Charleston, WV, USA; ^2^Department of Medicine, Rapides Regional Medical Center, Alexandria, LA, USA; ^3^Department of Medicine, Jinnah Medical & Dental College, Karachi, Pakistan

## Abstract

Sarcoidosis is an idiopathic, chronic, multisystem, granulomatous, inflammatory disease involving almost all organs. Sarcoidosis can occur with an atypical presentation of hepatosplenic involvement, like in the case of our patient. In this case report, we present a rare case of extrapulmonary sarcoidosis with isolated involvement of the liver and spleen in a 39-year-old Caucasian female. There is a possibility of this isolated involvement of an organ in the complete absence of pulmonary disease, which makes the diagnosis of sarcoidosis very difficult as it is usually not suspected. Ultrasound and CT are important in ruling out other differential diagnoses, but a definitive diagnosis is possible only on histological examination, differentiating sarcoid lesions from *tuberculosis*, primary biliary cirrhosis, metastasis, malignancy, and other granulomatous infections or diseases. Hence, the most credible criterion for diagnosis remains histology. After diagnosis, regular follow-up for systemic manifestations is recommended. Asymptomatic patients with hepatosplenic sarcoidosis have a good prognosis without any medical intervention, while patients with abnormal labs or symptoms must commence treatment.

## 1. Introduction

Sarcoidosis is a chronic, idiopathic, multisystem, noncaseous, granulomatous disease [1, 2]. The prevalence is highest in African Americans and observed mostly in young adults between 20 and 40 years of age [1]. More than 90% of cases show lung involvement, thus making chest radiography key for diagnosis [2]. While lungs are predominately affected in sarcoidosis, principally, every organ can get involved, including the skin, eye, and abdominal viscera, giving a much-varied presentation clinically [2]. While the literature reports extrapulmonary manifestations both in the presence and in the absence of pulmonary disease, isolated cases of extrapulmonary sarcoidosis is still a rare entity with only 10% cases reported [2]. Up to one-third of patients with sarcoidosis have a relapsing disease course causing severe organ dysfunction [2].

The gastrointestinal tract is very rarely the primary site in sarcoidosis; however, reported cases have commonly involved the liver and spleen in 80% of the cases but are usually asymptomatic [1, 3]. Isolated involvement in the absence of lung disease is infrequent and reported in only 13% of the systemic sarcoidosis cases [1]. If symptomatic, hepatic sarcoidosis presents as hepatosplenomegaly, raised liver enzymes, and intrahepatic cholestasis, which, in long-standing cases, leads to portal hypertension [1]. Splenic sarcoidosis is usually asymptomatic and may present with vague systemic features with frequently reported symptoms, including fatigue, fever, weight loss, and night sweats [2, 3]. In patients with a normal chest X-ray, extrapulmonary sarcoidosis has been identified and monitored using modalities like ultrasound, chest computed tomography (CT), gallium scintigraphy, magnetic resonance imaging (MRI), and positron emission tomography (PET) [4].

Routine lab investigations are futile; however, peripheral lymphopenia with CD4 depletion, raised serum angiotensin-converting enzyme (s-ACE), lysozyme, hypercalcemia, and hypercalciuria may help narrow differentials [2]. Criteria for diagnosing systemic sarcoidosis require symptoms showing the involvement of at least two organ systems, with supporting histology showing noncaseating granulomas, and valid exclusion of the possibility of any other granulomatous diseases [1].

Due to the rarity of exclusive extrapulmonary manifestations of sarcoidosis, especially involving the gastrointestinal tract, there are very scarce literature data published on diagnosis, management, and clinical course of the disease [1]. We present a case of isolated extrapulmonary gastrointestinal sarcoidosis with hepatic and splenic manifestations but without the signs of involvement of any other organ.

## 2. Case Presentation

A 39-year-old Caucasian female presented with complaints of intermittent nausea, vomiting, and abdominal pain for two months associated with weight loss of 30 pounds. The vomiting was nonbilious, and she also had reduced oral intake secondary to nausea. On examination, her vitals were a blood pressure of 100/60 mm Hg, pulse rate of 120/min, respiratory rate of 18/min, afebrile, and oxygen saturation of 98% in room air. She had no palpable lymph nodes with unremarkable cardiac and respiratory exams but had epigastric and right upper quadrant tenderness; the spleen was palpable 3 cm below the costal margin, and the liver span could not be assessed due to tenderness (Murphy's sign was positive).

**Figure 1:**
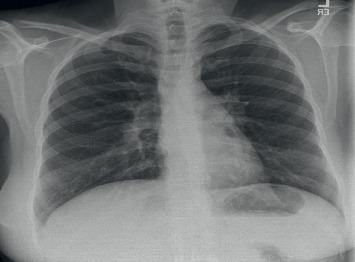
Chest X-ray showing no abnormality or masses.

Laboratory data identified anemia with a normal white blood cell count (WBC). They also demonstrated lactic acidosis of 6.4 mg/dl, alkaline phosphatase of 451 units/l, albumin 2.7 gm/dl, and the ionized calcium to be 1.62 meq (Table 1). The chest X-ray was normal, appearing with no remarkable findings (Figure 1); however, due to concern for cholecystitis, ultrasound of the abdomen was performed, and it reported that the patient had cholelithiasis with the heterogeneous liver.

**Figure 2:**
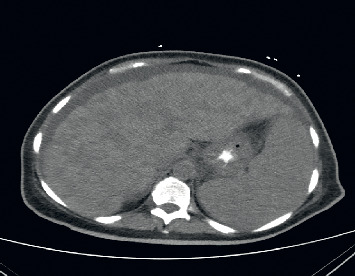
Computed tomography (CT) abdomen showing hepatosplenomegaly, numerous hypodensities in the liver.

**Figure 3:**
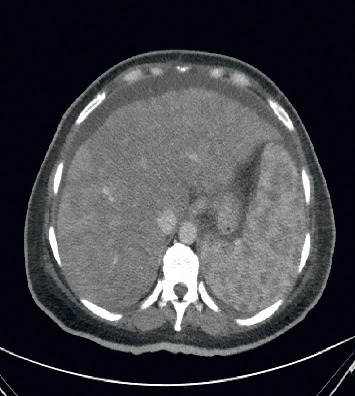
Computed tomography of the abdomen with contrast showing hepatosplenomegaly, numerous hypodensities in the spleen and ascites.

**Figure 4:**
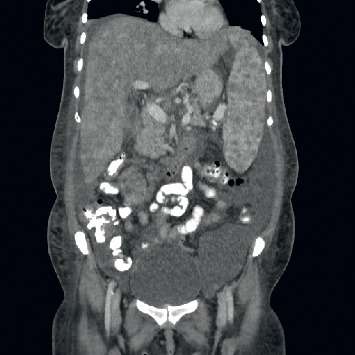
Computed tomography of the pelvis with contrast showing densities in the spleen.

Computed tomography of the abdomen with contrast (Figures 2–4) identified hepatosplenomegaly, numerous small density lesions in the liver and spleen. Further workup included paracentesis with a serum ascites albumin gradient (SAAG) >1 with fluid albumin <1 gm %, 40 WBCs, and negative Gram stain and cytology for malignant cells.

An upper gastrointestinal biopsy was performed to rule out peptic ulcer disease/malignancy; however, it showed chronic inflammatory changes. A hepatobiliary iminodiacetic acid (HIDA) scan had ruled out cholecystitis. An endoscopic retrograde cholangiopancreatography was performed, and it ruled out cholecystitis and showed cholelithiasis and nonobstructing bile duct without any evidence of any mass. A special set of labs was sent to rule out other etiologies of infiltrative disease (Table 2), which all came back negative.

**Figure 5:**
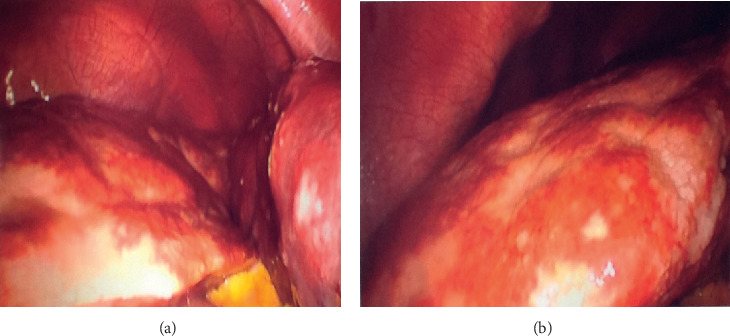
Exploratory laparoscopy showing infiltrative lesions.

**Figure 6:**
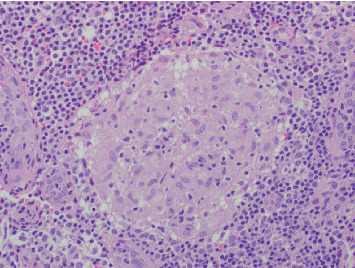
Histopathology showing noncaseating granulomas.

Finally, exploratory laparoscopy (Figure 5) was performed to reveal the characteristics of the infiltrative lesions. It revealed splenomegaly with small punctate lesions, hepatomegaly, and large intra-abdominal ascites, which was thick and proteinaceous. Histopathology identified noncaseating granulomas along with changes of steatohepatitis (Figure 6).

Based on the findings of histopathology and elevated calcium levels, a diagnosis of extrapulmonary sarcoidosis was established. The patient was started on steroid therapy and improved. Hypercalcemia improved slightly but remained persistent above normal (Table 3). She was followed up as an outpatient in two to three weeks, and her hypercalcemia had resolved. No recurrence of disease was reported at that follow-up visit.

## 3. Discussion

Sarcoidosis is characterized by noncaseating granulomas formed by the accumulation of inflammatory cells [5]. The sarcoid granuloma is a focal, chronic inflammatory reaction that contains epitheloid cells, giant cells, and CD4+ T cells in its center and CD8+ T-lymphocytes and B-lymphocytes at its periphery [6]. Granulomas also contain cytoplasmic inclusions, including asteroid bodies, Schaumann's bodies, calcium oxalate crystals, and Hamazaki-Wesenberg bodies [6, 7].

The extent of granuloma infiltration is positively associated with the amount of various inflammatory mediators released [8]. It can affect any organ, but most frequently involves the lungs (90%), lymph nodes (75%), eye (25%), and skin (25%) [1]. It primarily affects young adults between 25 and 40 years of age [5]. However, a second peak at 50 to 65 years of age is seen in about 30% of cases, showing gender predominance of females [5].

The literature records an approximate of 30% of cases of sarcoidosis in extrapulmonary locations, most common of which was abdomen (liver, spleen, biliary tree, peritoneum, and lymph nodes), with only 2% presenting as isolated cases without a concomitant thoracic disease [5]. Another atypical presentation of sarcoidosis is described by McGee and Minagar, who report the Heerfordt syndrome characterized by uveitis, bilateral parotid gland enlargement, and often facial nerve palsy [9]. This syndrome was first described by a Danish ophthalmologist Tamme et al. [10]. Caucasians with sarcoidosis were believed to be frequently associated with unregulated calcium metabolism, especially during active disease, resulting in hypercalciuria, hypercalcemia, and nephrolithiasis with renal impairment [11, 12]. This is believed to occur due to an increase in the activity of 1 − *α* hydroxylase in sarcoid alveolar macrophages that are responsible for changing 25-hydroxyvitamin D to 1, 25-dihydroxy vitamin D (the active form of vitamin D) [11, 12]. The incidence of hypercalcemia in sarcoidosis is widely variable, ranging from 2% to 63% [11]. Mild hypercalcemia is initially treated conservatively by restricting dietary calcium and sunlight exposure and increasing fluid intake [11]. Alternatively, if the serum calcium is >11 mg/dL or if the serum creatinine is raised or if the patient has nephrolithiasis, medical therapy is initiated [11]. Prednisone is commenced at an initial dose of 20–40 mg/day, which can rapidly lower serum calcium in 3–5 days [11, 12].

As per the diagnostic criteria, sarcoidosis is a systemic granulomatous disease that should involve several organs, and the involvement of a single organ will not form a diagnosis [11]. An exception to this is in the case of pulmonary involvement, where a physician may rightfully label a case of sarcoidosis with lung biopsy confirming the presence of noncaseous granulomatous inflammation of idiopathic origin along with a chest radiograph showing bilateral hilar adenopathy or a gallium 67 scan showing positive for hilar lymph nodes [11]. In the absence of pulmonary involvement, a diagnosis of sarcoidosis is made very vigilantly.

Hepatic sarcoidosis occurs in 1–40 per 100,000 cases [5]. However, this is believed to be underestimated as autopsy specimens show hepatic parenchyma involvement in 50%–80% of sarcoidosis cases [5]. Systemic symptoms reported include fever, weight loss, asthenia, jaundice, itching, anorexia, and abdominal pain [2, 3]. Hepatomegaly is seen in 5%–15% of patients; however, about 1% of patients have also reported portal hypertension with variceal bleeding, hepatopulmonary syndrome, and cirrhosis resulting in liver failure [2, 11]. Deranged liver function test with serum alkaline phosphatase levels being elevated in 90% of patients, and serum transaminases are elevated in 50%–70% of cases, but less in severity than alkaline phosphatase [11]. Hepatic nodules, which are less commonly seen than splenic nodules, have been reported in less than 5% cases, but were described as discrete, multiple, ranging in size of 0.6 cm–2.0 cm in diameter with a tendency of confluence, and of low attenuation, requiring intravenous contrast for visualization [2, 5, 11]. Differentials for low attenuation liver nodules include infections, metastasis, and lymphoma. The granulomatous lesions are present in the portal and periportal spaces of liver sinuses, demonstrating a uniform stage of maturation [5]. They can be seen as round or oval hypoechoic nodules on ultrasound, hypodense nodules on CT, and hypointense nodules on gadolinium-enhanced T1-weighted MRI [5].

ACE has been shown to be raised in 60% of cases, while hypercalcemia or hypercalciuria has been reported in 10–40% of patients [1, 2]. Although ACE levels may be used as a quick diagnostic tool, it has a high positive and negative predictive value of 84% and 74%, respectively, therefore lacking sensitivity and specificity [8]. A slight rise in the CA 19-9 level may indicate cholestasis [1].

Diagnosis is highly dependent on a compatible clinical presentation and radiographic findings; however, definitive diagnosis is only established with the histological presence of epithelioid noncaseating granuloma of the lymph node, lung, the or organ's specimen [2]. As granulomas of sarcoidosis exhibit no distinct histological features to help distinguish them from other granulomatous diseases, the latter must be excluded [2]. The common ones to be considered are neoplasia (lymphoma and solid tumors), autoimmune disease (Wegener's granulomatosis, primary biliary cirrhosis), farmer's lung disease, drug reactions, occupational and environmental exposure, and infections [2]. Thence, culture and stains for mycobacteria and fungi must always be ordered when considering sarcoidosis as a differential [2].

Treatment is based on the symptoms and severity of the disease [2]. The exclusive presence of noncaseating granulomas in the absence of abnormalities of liver function tests requires no treatment [2]. In symptomatic cases, first-line treatment remains glucocorticoids, which aids in the improvement of symptoms and laboratory reports but does not affect disease progression [2]. Prednisone at 30–60 mg/day has shown to alleviate symptoms, regulates the serum alkaline phosphatase level, and improves hepatomegaly [11]. Steroid alternatives such as azathioprine, methotrexate, and hydroxychloroquine are exclusively used in steroid-refractory disease [2]. While both azathioprine and methotrexate are possibly hepatotoxic, the former has been preferred [1, 4]. In advanced disease of the liver, transplantation is curative [1, 2].

Infrequently, hepatic sarcoidosis has led to a chronic cholestasis syndrome presenting with pruritis, jaundice, hepatomegaly, and markedly raised serum alkaline phosphatase and cholesterol [11]. Histologically, this can be seen as granulomas progressively and slowly destroying the bile ducts, mimicking histology of primary biliary cirrhosis [11]. The resultant ductopenia from this granulomatous cholangitis appears to be responsible for the chronic cholestasis established [11]. The sarcoid granulomas may also cause perisinusoidal block and occlude intrahepatic portal vein branches leading to portal hypertension, which has an incidence of 3% in hepatic sarcoidosis [11]. This may lead to esophageal or gastric variceal bleeding and death [11]. Seldom, patients with liver sarcoidosis may also develop the Budd–Chiari syndrome [11]. The symptoms associated with the chronic cholestatic syndrome are often severe and require immediate intervention [11]. In case of failure of treatment or further progression of the cholestatic syndrome, Urdodeoxycholic acid at a dose of 10 mg/kg/day has been successfully used to improve liver function tests and hepatic cholestasis symptoms [1, 11].

Portal hypertension seen in hepatic sarcoidosis may also be permanent if a resultant of fibrotic changes from biliary fibrosis or cirrhosis, which will, in turn, be unresponsive to corticosteroids or any other intervention being used to treat sarcoidosis [11]. However, hepatomegaly and liver function tests will improve with steroids [11]. Alternatively, the sarcoidosis resultant portal hypertension may be treated like that of any other cause, that is, with intravenous octreotide or vasopressin and a Sengstaken–Blakemore tube for acute esophageal or gastric variceal bleeding. Sclerotherapy of varices, *β* blockers, splenorenal, or transjugular intrahepatic portal-systemic shunt (TIPS), a splenectomy can also be used, or for refractory cases, a liver transplant is curative [1, 11]. It is advisable to initiate a corticosteroid trial before liver transplantation in patients with chronic liver failure from sarcoidosis [11].

Splenic sarcoidosis typically shows association with lung disease; however, 25%–33% of patients have also exhibited normal chest X-ray [5]. Splenic involvement in sarcoidosis on autopsy shows the greatest variation of 24%–59% [5]. Splenic sarcoidosis is usually asymptomatic but may occasionally incite left upper quadrant abdominal pain along with fever, malaise, and weight loss [2, 4, 5]. Laboratory investigations are normal, although some may develop anemia, thrombocytopenia, and neutropenia. An association between the ACE level and spleen size has been observed, with a study documenting s-ACE 3.1 times the upper limit in patients with splenic nodules [2]. Radiographic features of splenic lesions are not specific, and the differential diagnosis may include lymphoma, metastatic diseases, hemangioma, hematoma, abscess, hamartoma, and angiosarcoma [3]. Sarcoidosis of the spleen may be homogenous or nodular. Ultrasonography reveals splenomegaly in 33% of cases, with small hypoechoic nodules [4, 5]. CT shows hypodense confluent nodules on contrast and nodular lesions with low signal on all sequences of MRI [5]. T2-weighted MRI images serve as a marker of disease activity as hyperintense nodules appear in inflammation due to edema and increased vascular permeability [5]. Concomitant involvement of the liver and spleen has been recorded in 5–15% of patients [5]. Imaging features may mimic splenic neoplasms or infections; hence, a biopsy is indicated [3]. Needle biopsy may cause bleeding and lead to seeding; hence, laparoscopic splenectomy has been commonly used as an accepted surgical approach [3].

Most patients with splenic involvement of sarcoidosis will not necessitate treatment as a spontaneous resolution of splenomegaly is common (66%), especially when the spleen is <4 cm below the left costal margin [3, 11]. Medicinal therapy with prednisone, methotrexate, and/or antimalarial drugs has been reserved for symptomatic patients [2]. Indication for medical intervention includes abdominal pain due to splenomegaly, hypersplenism, functional asplenia, or rupture of the spleen [11]. As doses for corticosteroids are not standardized, its efficacy at reducing splenomegaly is not predictable [11]. In circumstances necessitating splenectomy, a prior corticosteroid trial is advised [11]. Indications for splenectomy include splenomegaly with discomfort, infarction, splenic rupture, or hypersplenism [11]. Generally, corticosteroid prednisone is the drug of choice with an initiating dose of 20–40 mg/day, which must be tapered to the lowest beneficial dose [11]. In case of failed attempts at tapering prednisone (or equivalent) to less than 10 mg/day within 3–6 months, alternative drugs must be considered [11].

To summarize, we report a case of systemic sarcoidosis with isolated extrapulmonary involvement of the liver and spleen. Identifying systemic locations radiographically is particularly tricky due to the wide array of imaging features found, which often overlap with that of other pathologies. It is important for the clinician to identify the pulmonary and extrapulmonary manifestations of the disease and recognize the radiographic findings in order to reduce morbidity and mortality [8]. Biopsy is an important diagnostic tool and can also be used to monitor disease in patients [1]. Mild asymptomatic disease has a good prognosis with spontaneous remission [1]. While basic guidelines for systemic manifestations of sarcoidosis are available, due to a lack of large-scale studies, a consistent treatment regimen has not been established [4]. Physicians must be vigilant with such patients and must manage them as prescribed by the guidelines or the clinician's personal experience.

## 4. Conclusions

This case highlights the diagnostic challenge that presents with isolated extrapulmonary sarcoidosis. Our case was of an extremely uncommon form of sarcoidosis as hepatosplenomegaly and hypercalcemia are rarely present without pulmonary involvement. The isolated hepatosplenic disease is a precursor for systemic disease; therefore, patients in this setting should be closely followed by their physicians for relapse.

## Figures and Tables

**Table 1:** Laboratory data.

Lab	Result
Lactic acid	6.4 mg/dl
Sodium	134 mmol/l
Potassium	3.4 mmol/l
Chloride	97 mmol/l
Bicarbonate	32 mmol/l
BUN/creatinine	13 mg/dl
Creatinine	1.2 mg/dl
Albumin	2.7 gm/dl
Ionized calcium	1.62 meq
Alkaline phosphatase	451 units/l
INR	1.37
White blood cells	3.4 mm^3^
Platelets	301 mm^3^
Hemoglobin	8.4 gm/dl

**Table 2:** Laboratory data to rule out other etiologies.

Laboratory entity	Results
Antinuclear antibody	Negative
Antismooth muscle antibody	Negative
Antimitochondrial antibody	Negative
CA 19-9	Not detected
CA-125	Not detected
Blood culture	Negative
Bacterial	
Fungal	
Anaerobic	
Histoplasma serology	Negative
Blastomyces serology	Negative
Cryptococcal serology	Negative
QuantiFERON gamma assay	Negative

**Table 3:** Laboratory results of calcium and vitamin-D on admission and discharge.

Laboratory entity	Admission	Discharge
Ionized calcium	1.62 meq	1.43 meq
Serum calcium	11.2 mg/dl	10.8 mg/dl
D25-hydroxy vitamin D	<4 units	116 units

## Data Availability

Data can be made available on request.
